# The Association Between Nut Consumption and Risk of Depressive Symptoms: A Meta-Analysis of Observational Studies

**DOI:** 10.3390/nu17243810

**Published:** 2025-12-05

**Authors:** Sohyun Kim, Hyogyeong Lee, Qiao-Yi Chen, Yooheon Park, NaNa Keum

**Affiliations:** Department of Food Science and Biotechnology, Dongguk University, SangYeong Building Room 543, Siksa-dong, Goyang-si 10326, Gyeonggi-do, Republic of Korea

**Keywords:** nut consumption, depressive symptoms, meta-analysis

## Abstract

**Background/Objectives:** Accumulating evidence suggests that dietary factors such as nuts may play a role in depressive symptoms. Yet, existing evidence regarding the relationship between nut consumption and depressive symptoms remains inconsistent. To clarify this association, we conducted a meta-analysis. **Methods:** PubMed and Embase were searched for observational studies on the relationship between nut consumption and depressive symptoms published up to September 2025. Summary relative risks (SRRs) and 95% confidence intervals (CIs) were estimated using the DerSimonian–Laird random effects model. **Results:** A total of seven observational studies, comprising 70,136 participants, were included. Higher nut consumption was significantly associated with a lower risk of depressive symptoms (SRRs = 0.75, 95% CIs, 0.67–0.85; *p* < 0.001, I^2^ = 15%, P_heterogeneity_ = 0.31). Compared to <1 time/week of nut consumption, the inverse relationship was significant for ≥3 times/week of nut consumption (SRRs = 0.75, 95% CIs = 0.63–0.89, *p* = 0.001, I^2^ = 0%, P_heterogeneity_ = 0.93), but not for 1 to <3 times/week of nut consumption (SRRs = 0.93, 95% CIs = 0.69–1.24, *p* = 0.62, I^2^ = 52%, P_heterogeneity_ = 0.10). **Conclusions:** Our meta-analysis of observational studies found that higher nut consumption was associated with a reduced risk of depressive symptoms, particularly when intake reached at least three servings per week. Further research, especially randomized controlled trials, is needed to understand the underlying mechanisms.

## 1. Introduction

Depression, also known as major depressive disorder, is one of the most prevalent mental disorders worldwide and a leading contributor to the global burden of disease [1,2]. As of 2021, approximately 332 million individuals of all ages, which accounts for about 4% of the global population, were estimated to have experienced depression [2]. Moreover, the global incidence of depression continues to rise. The age-standardized incidence rate (ASIR) was reported to be 4333.6 per 100,000 individuals worldwide in 2021, which represents a 15.6% increase compared with the ASIR in 1990 [3]. Projection further suggests that the ASIR of depression may increase by 95.6% by 2040 relative to the ASIR in 2021 [3]. This rising incidence of depression represents an alarming public health challenge [2,3], as depression is not only a major source of psychological distress but is also closely linked to adverse physical health outcomes [4,5,6,7]. Numerous studies have shown that individuals with depression have an increased risk of developing chronic diseases such as obesity, diabetes, hypertension, heart disease, and cancer, thereby contributing substantially to morbidity, premature mortality, and healthcare costs [5,6,7,8].

Beyond clinically diagnosed depression, subthreshold depression, defined by depressive symptoms that do not meet the full diagnostic criteria for clinical depression, is highly prevalent in the general population [9,10]. These subclinical depressive symptoms can substantially impair daily functioning, reduce quality of life, and strain interpersonal and social relationships [11,12]. Moreover, individuals with subthreshold depression are at elevated risk of progressing to major depressive disorder [9,10].

Given the high prevalence, and broad health consequences of both clinical and subclinical depression [2,4,5,6,7,9,10,11,12], there is a critical need to identify modifiable risk factors embedded in everyday life [13]. Dietary factors, as a fundamental component of daily behaviors, have been increasingly recognized as modifiable determinants of mental health [14,15]. Epidemiological studies indicate that healthy dietary patterns, such as the Mediterranean diet, are inversely associated with the risk of depressive symptoms [16,17]. The Mediterranean diet is characterized by high consumption of fruits, vegetables, whole grains, and nuts, alongside olive oil as the main fat source [18]. Among these components, nuts have attracted considerable attention due to their rich content of unsaturated fatty acids, vitamins, minerals, and bioactive compounds with anti-inflammatory and antioxidant properties, which may influence neurobiological pathways and reduce oxidative stress implicated in depression [19,20,21,22,23,24].

Despite growing interest, observational studies investigating the relationship between nut consumption and depressive symptoms have reported inconsistent results [25,26,27,28,29,30,31]. Previous systematic review was limited by the small number of available studies and the absence of a quantitative summary estimate, leaving the overall evidence inconclusive [32]. Therefore, we conducted a meta-analysis of observational studies to quantitatively assess the association between nut consumption and depressive symptoms.

## 2. Materials and Methods

This meta-analysis was performed and reported in accordance with the Preferred Reporting Items for Systematic reviews and Meta-Analysis (PRISMA) [33]. The PRISMA checklist is provided in Supplementary Table S1 [33]. Two authors (S.K. and H.L.) independently conducted the study search, study selection, and data abstraction. The disagreements between the two authors were resolved through discussion with N.K.

### 2.1. Study Search

Articles published up to September 2025 were identified through a comprehensive search of PubMed and Embase. Search strategies were constructed using MeSH terms (e.g., nuts, seeds, depression, and depressive disorder) and free-text terms (e.g., nut, seed, walnut, pistachio, hazelnut, peanut, depressive symptom, mental disorder), with the full syntax provided in Supplementary Table S2. The search was limited to English-language articles and human studies. Case reports, comments, letters and editorials were excluded, and no other restrictions were imposed.

### 2.2. Study Selection

**Figure 1:**
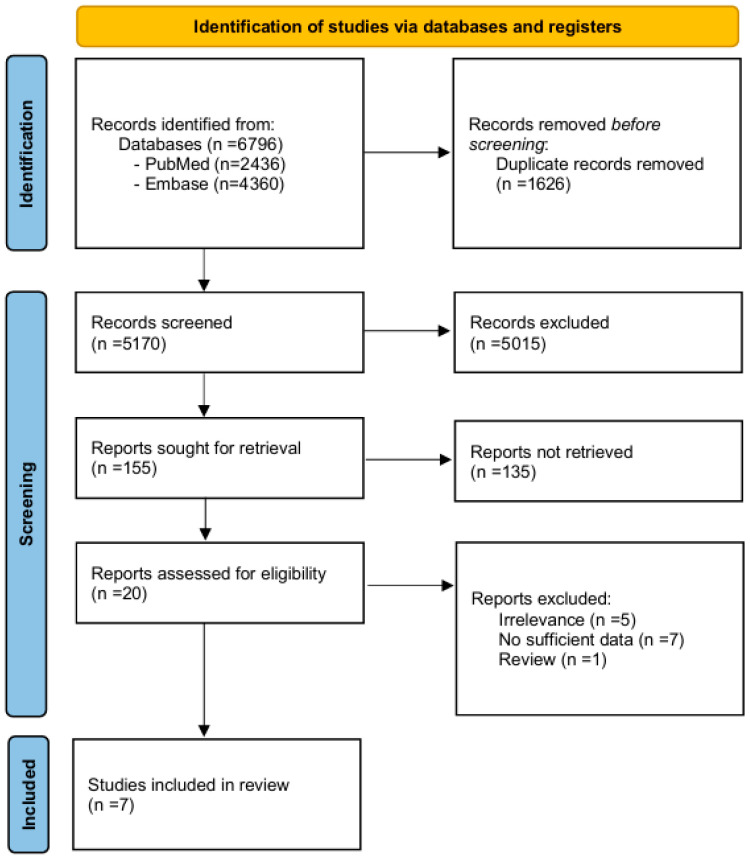
Flow diagram for study selection [33].

To be included in this meta-analysis, studies had to be observational studies that examined the relationship between any nut consumption and depressive symptoms. Abstracts, unpublished results, review articles, and studies conducted in special populations (e.g., pregnant women) were excluded. When both cohort and cross-sectional studies were available from the same study population, cohort studies were selected to minimize potential biases [27]. We also reviewed the reference lists of the selected articles [25,26,27,28,29,30,31] and previous systematic reviews [32] to check for any missing papers. After the screening process, a total of 7 studies [25,26,27,28,29,30,31] (3 cohort studies [26,27,30] and 4 cross-sectional studies [25,28,29,31]) were eligible for this meta-analysis. A detailed flow diagram of the study selection process is presented in Figure 1 [33].

To assess the quality of the studies, we used the Newcastle–Ottawa Scale (NOS) for cohort studies and the modified NOS for cross-sectional studies [34,35]. Both NOS and modified NOS consist of three categories (selection, comparability, and outcome), with a maximum score of 9 and 10, respectively [34,35]. Detailed results are presented in Supplementary Tables S3 and S4.

Of note, one study reported results from two independent cohort populations (Seniors-ENRICA-I and Seniors-ENRICA-II) separately [27]. Each cohort was considered an independent study and included individually in this meta-analysis.

### 2.3. Data Abstraction

From each study, the following information was extracted: first author, publication year, study design, the number of total participants and cases, key characteristics of the study population (e.g., age, sex, country), nut consumption (types, frequency, and portion size), assessment method of depressive symptoms (scales vs. clinical diagnosis), relative risk (RR), 95% confidence interval (CI), and confounding factors. Detailed data abstraction is shown in Supplementary Table S5.

### 2.4. Statistical Analyses

To evaluate the relationship between nut consumption and depressive symptoms, summary RRs (SRRs) and corresponding 95% CIs were calculated using the DerSimonian–Laird random effects model, which accounts for both within-study and between-study variability [36]. This approach was chosen to address potential heterogeneity arising from differences in study design, other methodological factors, and participant characteristics across the included studies [36]. Between-study variability was assessed using the I^2^ statistic, which measures the percentage of total variation across studies attributable to true heterogeneity rather than chance, and was used to assess the degree of heterogeneity among the studies [37]. I^2^ values were interpreted according to conventional thresholds, with approximately 25% indicating low heterogeneity, 50% indicating moderate heterogeneity, and 75% indicating high heterogeneity [38]. The statistical significance of heterogeneity was evaluated using Cochran’s Q test [39]. The potential for small-study effects (e.g., publication bias) was assessed visually using funnel plots and statistically using Egger’s test and Begg’s test [40]. To confirm the robustness of the primary findings, we conducted a sensitivity analysis by excluding each study at a time (i.e., leave-one-out analysis).

To explore the relationship between frequency of nut consumption and depressive symptoms, additional meta-analysis was conducted by the frequency of nut consumption (<3 servings per week vs. ≥3 servings per week). To investigate potential sources of heterogeneity in the relationship, subgroup analyses and meta-regressions were conducted according to pre-selected variables, including study design (cohort studies vs. cross-sectional studies), geographical location (Europe vs. the Americas vs. Asia), and mean age of study participants (<65 years vs. ≥65 years).

The two-sided *p*-value of <0.05 was regarded as statistically significant. All statistical analyses were performed using STATA 18.5 (StataCorp., College Station, TX, USA).

## 3. Results

### 3.1. Characteristics of Included Studies

After screening 6796 publications, a total of seven studies [25,26,27,28,29,30,31] (three cohort studies [26,27,30] and four cross-sectional studies [25,28,29,31]) were ultimately included in this meta-analysis. Overall, this meta-analysis comprised 70,136 participants, providing a substantial sample for evaluating the association between nut consumption and depressive symptoms. Main characteristics of the included studies are summarized in Supplementary Table S5. In brief, three studies were conducted in Europe [26,27,30], two studies were conducted in the Americas [28,29], and two studies were conducted in Asia [25,31], reflecting a geographically diverse study population. Five studies included participants aged <65 years [26,28,29,30,31] and two studies included those aged ≥65 years [25,27]. In terms of participant age, five studies had a mean age of <65 years [19,21,22,23,24], while two studies had a mean age of ≥65 years [18,20]. All studies adjusted for major confounders such as age, sex, body mass index, and drinking. In addition, six out of seven studies adjusted for physical activity, further strengthening the validity of the reported associations. Detailed information on the covariates adjusted for in each study is provided in Supplementary Table S5.

The results of the quality assessment are presented in Supplementary Tables S3 and S4. Among cohort studies [26,27,30], NOS scores ranged from 7 to 8 out of a maximum of 9 [34]. Similarly, among cross-sectional studies [25,28,29,31], modified NOS ranged from 7 to 8 out of a maximum of 10 [35]. Overall, the included studies had moderate-to-high methodological quality, making them appropriate for inclusion in the quantitative synthesis.

### 3.2. Primary Meta-Analysis of Nut Consumption and Risk of Depressive Symptoms

**Figure 2:**
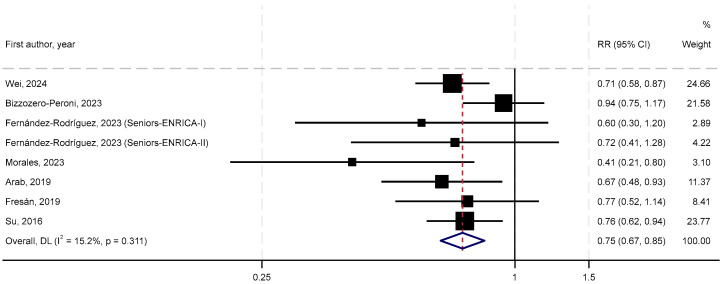
Meta-analysis of nut consumption and risk of depressive symptoms [25,26,27,28,29,30,31].

Figure 2 presents the forest plot for the primary meta-analysis of seven studies [25,26,27,28,29,30,31], including three cohort studies [26,27,30] and four cross-sectional studies [25,28,29,31]). The SRR of depressive symptoms for the highest vs. lowest nut consumption was 0.75 (95% CIs = 0.67–0.85, *p* < 0.001), with a low degree of heterogeneity (I^2^ = 15%, P_heterogeneity_ = 0.31). Evidence of small-study effects, such as publication bias, was suggested by visual asymmetry in the funnel plot (Supplementary Figure S1). However, Egger’s test (P_Egger_ = 0.15) and Begg’s test (P_Begg_ = 0.54) did not indicate statistical significance of small-study effects. In the leave-one-out sensitivity analysis, the overall association remained robust and was largely unchanged.

**Figure 3:**
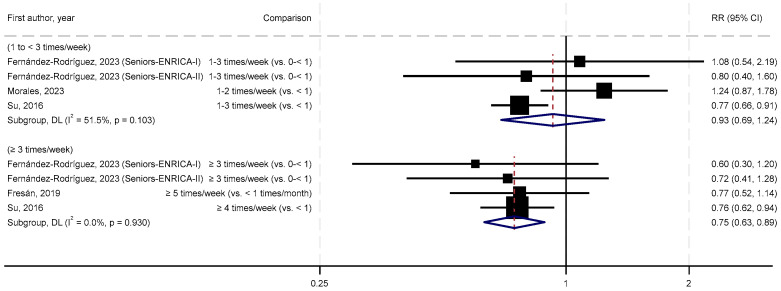
Meta-analysis by frequency of nut consumption [27,28,30,31].

Figure 3 presents the forest plot for the meta-analysis stratified by frequency of nut consumption. For consumption of 1 to <3 times/week vs. <1 time/week, the SRR was 0.93 (95% CIs = 0.69–1.24, *p* = 0.62, *n* = 3 studies), with a moderate degree of heterogeneity (I^2^ = 52%, P_heterogeneity_ = 0.10). For consumption of ≥3 times/week vs. <1 time/week, the SRR was 0.75 (95% CIs = 0.63–0.89, *p* = 0.001, *n* = 3 studies), with no evidence of heterogeneity (I^2^ = 0%, P_heterogeneity_ = 0.93).

### 3.3. Subgroup Meta-Analysis

**Figure 4:**
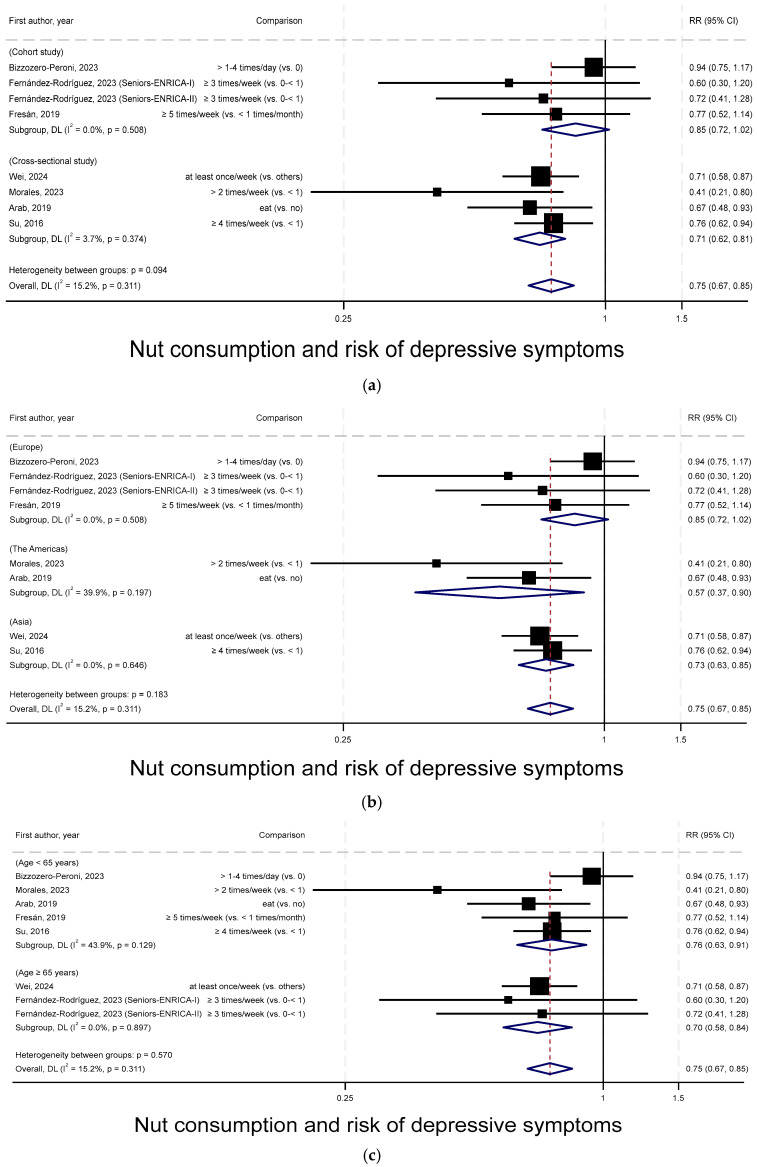
Subgroup meta-analysis by (**a**) study design, (**b**) geographic location, (**c**) mean age of participants [25,26,27,28,29,30,31].

Figure 4a presents the subgroup meta-analysis forest plot stratified by study design. Among cohort studies, the SRR was 0.85 (95% CIs = 0.72–1.02, *p* = 0.08, *n* = 3 studies), with no evidence of heterogeneity (I^2^ = 0%, P_heterogeneity_ = 0.51). Among cross-sectional studies, the SRR was 0.71 (95% CIs = 0.62–0.81, *p* < 0.001, *n* = 4 studies), with a low degree of heterogeneity (I^2^ = 4%, P_heterogeneity_ = 0.37). Heterogeneity in SRRs by study design was not statistically significant (P_heterogeneity_ = 0.15).

Figure 4b presents the subgroup meta-analysis forest plot stratified by geographic location. Among studies conducted in European countries, the SRR was 0.85 (95% CIs = 0.72–1.02 *p* = 0.08, *n* = 3 studies), with no evidence of heterogeneity (I^2^ = 0%, P_heterogeneity_ = 0.51). Among studies conducted in countries across the Americas, the SRR was 0.57 (95% CIs = 0.37–0.90, *p* < 0.05, *n* = 2 studies), with a moderate degree of heterogeneity (I^2^ = 40%, P_heterogeneity_ = 0.20). Among studies conducted in Asian countries, the SRR was 0.73 (95% CIs = 0.63–0.85, *p* < 0.001, *n* = 2 studies), with no evidence of heterogeneity (I^2^ = 0%, P_heterogeneity_ = 0.65). Heterogeneity in SRRs by geographic location was not statistically significant (P_heterogeneity_ = 0.31).

Figure 4c presents the subgroup meta-analysis forest plot stratified by mean age of study participants. Among studies with mean age of <65 years, the SRR was 0.76 (95% CIs = 0.63–0.91, *p* < 0.05, *n* = 5 studies), with a moderate degree of heterogeneity (I^2^ = 44%, P_heterogeneity_ = 0.13). Among studies with mean age of age ≥65 years, the SRR was 0.70 (95% CIs = 0.58–0.84, *p* < 0.001, *n* = 2 studies), with no evidence of heterogeneity (I^2^ = 0%, P_heterogeneity_ = 0.90). Heterogeneity in SRRs by mean age of study participants was not statistically significant (P_heterogeneity_ = 0.50).

## 4. Discussion

In this meta-analysis of observational studies, higher nut consumption was modestly but significantly associated with a decreased risk of depressive symptoms. The inverse association was significant for high nut consumption (≥3 times/week) but not for moderate nut consumption (1 to <3 times/week), compared to low consumption (<1 time/week). In subgroup analyses by study design and geographic location, a modestly lower risk of depressive symptoms associated with higher nut consumption was observed in cross-sectional studies (but not in cohort studies) and in studies conducted in the Americas or Asia (but not in Europe). A modest but significant inverse association remained consistent across subgroups defined by mean age of study participants.

The potential protective effects of nut consumption against depressive symptoms may be attributed to the diverse bioactive nutrients found in nuts. Nuts are rich sources of anti-inflammatory and antioxidant compounds, including omega-3 fatty acids, magnesium, vitamin E, flavonoids, and lignans [19,20]. These nutrients, by reducing inflammation, oxidative stress, and mitochondrial dysfunction—key processes linked to the development of depressive symptoms—may enhance neuroplasticity and neurogenesis [21,22,23], which are essential for emotional regulation and mental well-being [24]. In addition, nuts are a good dietary source of tryptophan [41]. Once absorbed, tryptophan crosses the blood–brain barrier and is converted into serotonin, a key neurotransmitter involved in mood regulation [42]. Dietary fiber in nuts also promotes the growth of beneficial gut bacteria and enhances microbial diversity [43,44], which may influence tryptophan metabolism and its availability for serotonin production in the brain [42]. Therefore, the potential role of nuts in modulating depressive symptoms through tryptophan–serotonin pathways or gut microbiota is biologically plausible, but direct evidence linking nut consumption to brain serotonin levels in humans remains limited and warrants further investigation [41,42,43,44].

In our meta-analysis, high nut consumption (≥3 times/week), but not moderate nut consumption (1 to <3 times/week), was associated with a reduced risk of depressive symptoms, suggesting the possibility of a threshold effect. This finding is consistent with previous evidence indicating that meaningful health benefits and disease prevention often emerge only when intake levels of specific nutrients or foods exceed certain thresholds [45]. For example, dietary fiber—a key component of nuts and a well-established determinant of gut and metabolic health [43,44]—was shown to reduce depressive symptoms only when consumed at or above 7.8 g per 1000 kcal per day [46].

An inverse association between nut intake and depressive symptoms was observed in the meta-analysis of cross-sectional studies; however, this association did not reach statistical significance in the meta-analysis of cohort studies, which provide more robust evidence for causal inference [47,48]. The observed discrepancy can be explained by several methodological factors. First, the definition of depressive outcomes differed across study designs. Cohort studies relied on physician-diagnosed depression, likely capturing moderate to severe cases [26,27,30], whereas cross-sectional studies used self-reported symptom scales [25,28,29,31], which are more sensitive to milder or subclinical symptoms [9]. This suggests that nut consumption may have a more pronounced effect on less severe depressive states. Second, reverse causation in cross-sectional studies could lead to a spurious association [49]. However, this is unlikely, as individuals with depressive symptoms tend to prefer sweet foods over nuts as a form of self-medication [50].

In our meta-analysis by geographic location, an inverse association was observed among studies conducted in countries in the Americas or Asia, but not among studies conducted in European countries. Consistent with our earlier suggestion that nut consumption may have a more pronounced effect on less severe depressive states, studies from the Americas and Asia primarily relied on self-reported symptom scales that capture mild or subclinical depressive symptoms [25,28,29,31], whereas European studies more often used physician diagnoses, which reflect moderate to severe clinical depression [26,27,30]. Additionally, regional differences in the types of nuts consumed may contribute to the observed variation. For example, compared to European countries, American or Asian countries tend to consume more peanuts and fewer hazelnuts [51], and peanuts contain higher levels of tryptophan than hazelnuts (0.25 vs. 0.19 g per 100 g) [52].

In our meta-analysis, an inverse association between nut consumption and depressive symptoms was observed in studies of both younger (mean age < 65 years) and older (mean age ≥ 65 years) adults, suggesting that the potential mental health benefits of nuts may operate through age-independent biological pathways. For instance, anti-inflammatory and antioxidant compounds abundant in nuts may help reduce depressive symptoms across age groups, aligning with evidence that higher dietary inflammation and lower antioxidant intake were consistently associated with a higher depression risk in both young and older adults [19,20,53,54,55,56]. Therefore, nut consumption could serve as a broadly applicable dietary strategy throughout adulthood [53,54,55,56], including among older populations who are often vulnerable to age-related physiological changes and depression [57]. Supporting this possibility, the PREDIMED randomized controlled trial, which included men aged 55–80 years and women aged 60–80 years, reported that participants assigned to the Mediterranean diet supplemented with mixed nuts had the lowest incidence of depression compared with participants assigned to the control diet or to the Mediterranean diet supplemented with extra-virgin olive oil [58].

Our study has several limitations. First, as all included studies were observational, bias due to unmeasured or residual confounding cannot be ruled out [59]. For instance, nut intake may reflect broader healthy lifestyle patterns; however, not all included studies adjusted for overall dietary quality, and considerable variability existed in the selection and modeling of dietary covariates, which may have influenced the observed associations. Second, many studies assessed depressive symptoms using participants’ self-report, which may introduce measurement error [60]. However, this error is likely to be random with respect to nut intakes, as nut consumption was assessed alongside diverse dietary factors [61]. With random measurement errors typically biasing results toward the null, the observed association in our study is unlikely due to this error [62]. Third, the definition of nuts varied across studies, with “mixed nut” exposure including up to 20 types in one study [27] and as few as 7 in the other study [31]. As a result, analysis by individual nut type could not be performed, although some nuts may offer greater benefits than others. Fourth, although *p*-values from Egger’s and Begg’s test were >0.05, given the limited number (<10 studies) of available studies for this meta-analysis, the tests were low-powered and we cannot rule out potential publication bias. Nevertheless, to our knowledge, this is the first meta-analysis that quantitatively summarized the relationship between nut consumption and the risk of depressive symptoms.

## 5. Conclusions

In our meta-analysis of observational studies, higher nut consumption was associated with a lower risk of depressive symptoms, particularly when intake reached at least three servings per week. Future randomized controlled trials are warranted to confirm this association and to elucidate the underlying mechanisms. In addition, future epidemiological studies incorporating a wider range of intake categories and which differentiate among various types of nuts are needed to evaluate the dose–response relationships and to clarify the optimal level and type of nut consumption most strongly associated with reduced depressive symptoms.

## Data Availability

The original contributions presented in this study are included in the article/Supplementary Material. Further inquiries can be directed to the corresponding author.

## Supplementary Materials

The following supporting information can be downloaded at https://www.mdpi.com/article/10.3390/nu17243810/s1, Figure S1: Funnel Plot; Table S1: PRISMA Checklist; Table S2: Database Search Strategy; Table S3: NOS for Cohort Studies; Table S4: Modified NOS for Cross-Sectional Studies; Table S5: Characteristics of Studies.
