# Supplementary material for: The Association Between Nut Consumption and Risk of Depressive Symptoms: A Meta-Analysis of Observational Studies
Source: Nutrients. 2025 Dec 5;17(24):3810. doi: 10.3390/nu17243810 (PMC12736318; doi:10.3390/nu17243810)

Supplementary Table S1. PRISMA Checklist [33]

Section and Topic	Item #	Checklist item	Location where item is reported
**TITLE**			
Title	1	Identify the report as a systematic review.	1
**ABSTRACT**			
Abstract	2	See the PRISMA 2020 for Abstracts checklist.	1
**INTRODUCTION**			
Rationale	3	Describe the rationale for the review in the context of existing knowledge.	1-2
Objectives	4	Provide an explicit statement of the objective(s) or question(s) the review addresses.	1-2
**METHODS**			
Eligibility criteria	5	Specify the inclusion and exclusion criteria for the review and how studies were grouped for the syntheses.	2-3
Information sources	6	Specify all databases, registers, websites, organisations, reference lists and other sources searched or consulted to identify studies. Specify the date when each source was last searched or consulted.	2-3
Search strategy	7	Present the full search strategies for all databases, registers and websites, including any filters and limits used.	2-3
Selection process	8	Specify the methods used to decide whether a study met the inclusion criteria of the review, including how many reviewers screened each record and each report retrieved, whether they worked independently, and if applicable, details of automation tools used in the process.	2
Data collection process	9	Specify the methods used to collect data from reports, including how many reviewers collected data from each report, whether they worked independently, any processes for obtaining or confirming data from study investigators, and if applicable, details of automation tools used in the process.	2-4
Data items	10a	List and define all outcomes for which data were sought. Specify whether all results that were compatible with each outcome domain in each study were sought (e.g. for all measures, time points, analyses), and if not, the methods used to decide which results to collect.	4
	10b	List and define all other variables for which data were sought (e.g. participant and intervention characteristics, funding sources). Describe any assumptions made about any missing or unclear information.	4
Study risk of bias assessment	11	Specify the methods used to assess risk of bias in the included studies, including details of the tool(s) used, how many reviewers assessed each study and whether they worked independently, and if applicable, details of automation tools used in the process.	3
Effect measures	12	Specify for each outcome the effect measure(s) (e.g. risk ratio, mean difference) used in the synthesis or presentation of results.	4
Synthesis methods	13a	Describe the processes used to decide which studies were eligible for each synthesis (e.g. tabulating the study intervention characteristics and comparing against the planned groups for each synthesis (item #5)).	2-4
	13b	Describe any methods required to prepare the data for presentation or synthesis, such as handling of missing summary statistics, or data conversions.	4
	13c	Describe any methods used to tabulate or visually display results of individual studies and syntheses.	2-4
	13d	Describe any methods used to synthesize results and provide a rationale for the choice(s). If meta-analysis was performed, describe the model(s), method(s) to identify the presence and extent of statistical heterogeneity, and software package(s) used.	4
	13e	Describe any methods used to explore possible causes of heterogeneity among study results (e.g. subgroup analysis, meta-regression).	4
	13f	Describe any sensitivity analyses conducted to assess robustness of the synthesized results.	4
Reporting bias assessment	14	Describe any methods used to assess risk of bias due to missing results in a synthesis (arising from reporting biases).	4
Certainty assessment	15	Describe any methods used to assess certainty (or confidence) in the body of evidence for an outcome.	_
**RESULTS**			
Study selection	16a	Describe the results of the search and selection process, from the number of records identified in the search to the number of studies included in the review, ideally using a flow diagram.	2-4

Section and Topic	Item #	Checklist item	Location where item is reported
	16b	Cite studies that might appear to meet the inclusion criteria, but which were excluded, and explain why they were excluded.	2-3
Study characteristics	17	Cite each included study and present its characteristics.	4-5
Risk of bias in studies	18	Present assessments of risk of bias for each included study.	5
Results of individual studies	19	For all outcomes, present, for each study: (a) summary statistics for each group (where appropriate) and (b) an effect estimate and its precision (e.g. confidence/credible interval), ideally using structured tables or plots.	4-7
Results of syntheses	20a	For each synthesis, briefly summarise the characteristics and risk of bias among contributing studies.	4-7
	20b	Present results of all statistical syntheses conducted. If meta-analysis was done, present for each the summary estimate and its precision (e.g. confidence/credible interval) and measures of statistical heterogeneity. If comparing groups, describe the direction of the effect.	4-7
	20c	Present results of all investigations of possible causes of heterogeneity among study results.	6-7
	20d	Present results of all sensitivity analyses conducted to assess the robustness of the synthesized results.	5
Reporting biases	21	Present assessments of risk of bias due to missing results (arising from reporting biases) for each synthesis assessed.	5
Certainty of evidence	22	Present assessments of certainty (or confidence) in the body of evidence for each outcome assessed.	_
**DISCUSSION**			
Discussion	23a	Provide a general interpretation of the results in the context of other evidence.	8-9
	23b	Discuss any limitations of the evidence included in the review.	9
	23c	Discuss any limitations of the review processes used.	9
	23d	Discuss implications of the results for practice, policy, and future research.	9
**OTHER INFORMATION**			
Registration and protocol	24a	Provide registration information for the review, including register name and registration number, or state that the review was not registered.	_
	24b	Indicate where the review protocol can be accessed, or state that a protocol was not prepared.	_
	24c	Describe and explain any amendments to information provided at registration or in the protocol.	_
Support	25	Describe sources of financial or non-financial support for the review, and the role of the funders or sponsors in the review.	10
Competing interests	26	Declare any competing interests of review authors.	10
Availability of data, code and other materials	27	Report which of the following are publicly available and where they can be found: template data collection forms; data extracted from included studies; data used for all analyses; analytic code; any other materials used in the review.	2-4

Supplementary Table S2. Database Search Strategy

PubMed	(Nuts[mesh] OR seeds[mesh] OR "Diet, Mediterranean"[Mesh] OR nut[tw] OR nuts[tw] OR seed[tw] OR seeds[tw] OR “mediterranean diet"[tw] OR Walnut*[tw] OR almond[tw] OR pecan*[tw] OR Brazil nut*[tw] OR pistachio*[tw] OR macadamia*[tw] OR cashew*[tw] OR hazelnut*[tw] OR peanut*[tw] OR pinenut*[tw] OR sunflower seed*[tw] OR legume*[tw] OR chestnut*[tw]) AND (depression[mesh] OR depressive disorder[mesh] OR depression[tw] OR depressive disorder[tw] OR depressive symptom*[tw] OR mental disorder*[tw] OR mood disorder*[tw] OR melancholia[tw]) NOT (Case Reports[ptyp] OR Comment[ptyp] OR Letter[ptyp] OR Editorial[ptyp]) NOT (animals[Mesh] NOT humans[Mesh])
Embase	(‘Nut’/exp OR ‘plant seed’/exp OR ‘Mediterranean diet’/exp OR ‘nut’:ti,ab OR ‘nuts’:ti,ab OR ‘seed’:ti,ab OR ‘seeds’:ti,ab OR ‘mediterranean diet’:ti,ab OR ‘Walnut*’:ti,ab OR ‘almond’:ti,ab OR ‘pecan*’:ti,ab OR ‘Brazil nut*’:ti,ab OR ‘pistachio*’:ti,ab OR ‘macadamia*’:ti,ab OR ‘cashew*’:ti,ab OR ‘hazelnut*’:ti,ab OR ‘peanut*’:ti,ab OR ‘pinenut*’:ti,ab OR ‘sunflower seed*’:ti,ab OR ‘legume*’:ti,ab OR ‘chestnut*’:ti,ab) AND (‘Depression’/exp OR ‘depress*’:ti,ab OR ‘depressive disorder’:ti,ab OR ‘depressive symptom*’:ti,ab OR ‘mental disorder*’:ti,ab OR ‘mood disorder*’:ti,ab OR ‘melancholia’:ti,ab) NOT (‘case report’/de OR ‘comment’/de OR ‘letter’/de OR ‘editorial’/de) NOT ([animals]/lim NOT [humans]/lim)

Supplementary Table S3. NOS for Cohort Studies [34]

	First author year [reference]			
	Bizzozero-Peroni 2023 [26]	Fernández-Rodríguez 2023 (Seniors-ENRICA-I) [27]	Fernández-Rodríguez 2023 (Seniors-ENRICA-II) [27]	Fresán 2019 [30]
Selection				
1. Representativeness of the exposed cohort	*	*	*	*
2. Selection of the non exposed cohort	*	*	*	*
3. Ascertainment of exposure	*	*	*	*
4. Demonstration that outcome of interest was not present at start of study	*	*	*	*
Comparability				
1. Comparability of cohorts on the basis of the design or analysis	**	**	**	**
Outcome				
1. Assessment of outcome				
2. Was follow-up long enough for outcomes to occur	*	*	*	*
3. Adequacy of follow up of cohorts		*		*
Score	7	8	7	8

Supplementary Table S4. Modified NOS for Cross-Sectional Studies [35]

	First author year [reference]			
	Wei 2024 [25]	Morales 2023 [28]	Arab 2019 [29]	Su 2016 [31]
Selection				
1. Representativeness of the sample	*		*	*
2. Sample size	*	*	*	*
3. Non-respondents		*		
4. Ascertainment of the exposure (risk factor)	*	**	**	**
Comparability				
1. The subjects in different outcome groups are comparable, based on the study design or analysis. Confounding factors are controlled.	**	*	*	**
Outcome				
1. Assessment of outcome	*	*	*	*
2. Statistical test	*	*	*	*
Score	7	7	7	8

Supplementary Table S5. Characteristics of Studies

First author Year Reference	Study design	Number of total participants	Number of cases	Mean age Female (%) Country	Nut consumption			Assessment method of depressive symptoms	RR (95% CI)	Confounding factors
					Types	Frequency	Portion size			
Wei 2024 [25]	cross-sectional	12,529	1,826	83.5 54% China	nut products	at least once/week (vs. others)	–	scales (Center for Epidemiologic Studies Depression (CES-D-10))	0.71 (0.58, 0.87)	age, gender, race, marital status, residence, education, occupation, body mass index, smoking, alcohol drinking, socioeconomic status, living arrangements, living preference, comorbidities, serious illness in the past 2 years, social/ leisure activity score, poor interviewer-rated health, hearing problems, visual impairment, functional limitation, cognitive impairment. physical exercise
Bizzozero-Peroni 2023 [26]	cohort	10,692	929	57.5 51% UK	mixed nuts	>1-4 times/day (vs. 0)	30g	clinical diagnosis	0.94 (0.75, 1.17)	sex, age, educational or professional qualification, body mass index, tobacco use, alcohol consumption, major dietary changes in the last five years, dietary pattern, physical activity, sleep duration, and total energy intake, loneliness and self-reported medical conditions diagnosed by a doctor
Fernández-Rodríguez 2023 [27]	cohort (Seniors-ENRICA-I)	1,363	95	71.4 53% Spain	mixed nuts	≥3 times/week (vs. 0-<1)	30g	clinical diagnosis	0.60 (0.30, 1.20)	sex, age, educational level and living alone, smoking status, alcohol consumption, sleep duration, physical inactivity, body mass index, Mediterranean diet adherence screen score, diabetes mellitus, cardiovascular disease, hypertension, obstructive sleep apnea syndrome, cancer at any site, and neurodegenerative disease
Fernández-Rodríguez 2023 [27]	cohort (Seniors-ENRICA-II)	1,323	63	71.4 46% Spain	mixed nuts	≥3 times/week (vs. 0-<1)	30g	clinical diagnosis	0.72 (0.41, 1.28)	sex, age, educational level and living alone, smoking status, alcohol consumption, sleep duration, physical inactivity, body mass index, Mediterranean diet adherence screener score, diabetes mellitus, cardiovascular

										disease, hypertension, obstructive sleep apnea syndrome, cancer at any site, and neurodegenerative disease
Morales 2023 [28]	cross-sectional	934	223	19 53% Chilean	nuts	>2 times/week (vs. <1)	–	scales (Depression Anxiety and Stress Scale (DASS-21))	0.41 (0.21, 0.80)	sex, age, maternal schooling level, body mass index, tobacco use, alcohol use, sedentary lifestyle, and physical activity
Arab 2019 [29]	cross-sectional	21,256	1,942	46 51% America	walnuts with other nuts	eat (vs. no)	–	scales (Patient Health Questionnaire (PHQ-9))	0.67 (0.48, 0.93)	age, sex, race, body mass index, smoking, alcohol use, income, marital status, and interaction between walnut consumption and sex
Fresán 2019 [30]	cohort	15,980	666	36.7 60% Spain	nuts	≥5 times/week (vs. <1 times/month)	–	clinical diagnosis	0.77 (0.52, 1.14)	sex, body mass index, total energy intake, physical activity during free time, time spent in sedentary activities, tobacco, marital status, the baseline self-perception of dependence, anxiety and competitiveness levels, stratified by age and year of entrance to the cohort, and the rest of the components of the score
Su 2016 [31]	cross-sectional	6,059	486	China 43.5 44%	mixed nuts	≥4 times/week (vs. <1)	–	scales (Self-Rating Depression Scale (SDS))	0.76 (0.62, 0.94)	age, sex, body mass index, smoking status, drinking status, physical activity, marital status, total energy intake, household incomes, employment status, educational levels, visiting friends, living alone, metabolic syndrome, and intake of eicosapentaenoic acid+ docosahexaenoic acid

Supplementary Figure S1. Funnel Plot

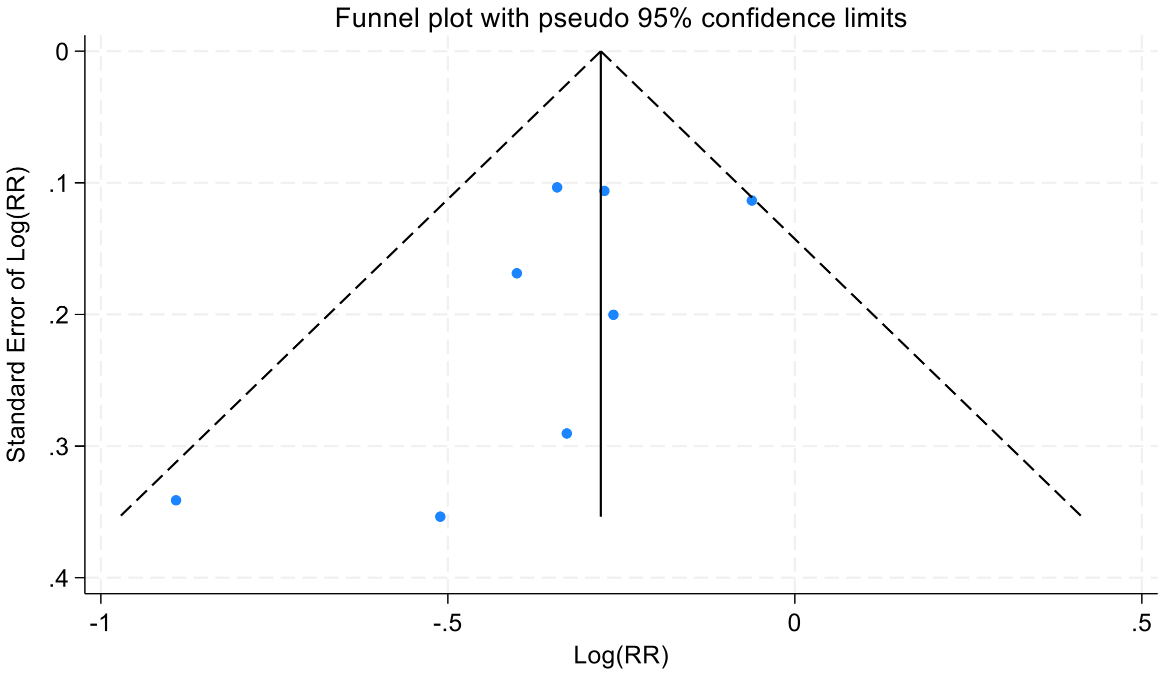

Supplement: Supplementary file 1 [file nutrients-17-03810-s001.zip › nutrients-3990395-supplementary.pdf]
